# The Correlated Risk Factors for Severe Liver Damage Among HIV-Positive Inpatients With Abnormal Liver Tests

**DOI:** 10.3389/fmed.2022.817370

**Published:** 2022-02-22

**Authors:** Sheng Liu, Ying Zhou, Yu Wang, Cheng Bo Li, Wen Wang, Xu Lu, Pei Liu, Qing Hai Hu, Ying Wen

**Affiliations:** ^1^Infectious Diseases Department, The First Affiliated Hospital of China Medical University, Shenyang, China; ^2^Key Laboratory of AIDS Immunology of Ministry of Health, Department of Laboratory Medicine, The First Affiliated Hospital of China Medical University, Shenyang, China

**Keywords:** acquired immune deficiency syndrome (AIDS), severe liver damage, antiretroviral therapy (ART), logistic regression model, risk factors

## Abstract

**Background:**

This study investigated the factors correlated with severe liver damage among HIV-infected inpatients.

**Methods:**

We retrospectively collected the first hospitalized HIV-infected patients in the Department of Infectious Disease of the First Affiliated Hospital of China Medical University from January 1, 2010, to December 31, 2019. We used multivariate logistic regression to identify the factors associated with severe liver damage.

**Results:**

A total of 493 patients with abnormal liver tests were recruited. Among 63 cases (12.8%) with severe liver injury, drug-induced liver injury (DILI) identified by the updated Roussel Uclaf Causality Assessment Method (RUCAM) score as the direct cause was found in 43 cases. Anti-tuberculosis drug (ATD) exposure [adjusted odds ratio (aOR) = 1.835, 95% confidence interval (CI): 1.031–3.268], cotrimoxazole exposure (aOR = 2.775, 95% CI: 1.511–5.096), comorbidity of viral hepatitis (aOR = 2.340, 95% CI: 1.161–4.716), alcohol consumption history (aOR = 2.392, 95% CI: 1.199–4.769), and thrombocytopenia (aOR = 2.583, 95% CI:1.127–5.917) were associated with severe liver injury (all *P* < 0.05).

**Conclusions:**

DILI was the predominant cause of severe liver damage, followed by hepatitis virus co-infection. For patients with alcohol consumption and thrombocytopenia, frequent monitoring of liver function tests should be considered.

## Background

Liver-related death is the common cause of non-acquired immune deficiency syndrome (AIDS)-related death, which is mainly due to decompensated cirrhosis and hepatocellular carcinoma among human immunodeficiency virus (HIV) patients co-infected with hepatitis B virus (HBV) or hepatitis C virus (HCV) (1). Liver-related death is occasionally associated with fulminant hepatic failure (FHF) caused by drug-induced liver injury (DILI) and hepatitis virus co-infection (2, 3). In a cohort of antiretroviral therapy (ART)-experienced individuals from high-income countries, approximately 14.5% of deaths were from liver-related causes (4). Liver enzyme elevation is common among HIV-infected inpatients, and 50% of these patients are asymptomatic (5). The prevalence of mild and moderate liver enzyme elevations associated with steatosis/steatohepatitis among ART-treated patients was much higher than that among ART-naïve patients, mainly due to increased body mass index (BMI) (6–8). The liver damage incidence in China was highly observed within 6–12 months after ART initiation (9). DILI frequently occurs in patients with older ART regimens (10). Hazardous alcohol consumption is also a risk factor for liver disease in HIV-infected populations (11). The liver is also a commonly involved site due to common systemic opportunistic infections (OIs), including Mycobacterium tuberculosis (MTB), non-tuberculosis mycobacteria (NTM), fungi, and cytomegalovirus (CMV) (10). Furthermore, hepatic TB-immune reconstitution inflammatory syndrome (IRIS) was also an etiology of liver damage shortly after ART (10).

The occurrence of severe liver events often resulted in liver-related hospital admissions, elevated risk of hepatic failure, and alteration in the medical care of other fatal illnesses. The alanine transaminase (ALT) was often the only index for monitoring liver disease in HIV-positive populations (12, 13). At the same time, multiple variables were already used to evaluate liver injury (6). The alkaline phosphatase (ALP) and gamma-glutamyl transferase (GGT) should be assessed in cholestasis. The total bilirubin (TBIL), direct bilirubin (DBIL), cholinesterase (CHE), serum albumin (ALB), prothrombin time (PT) and international normalized ratio (INR) should be assessed in liver failure or decompensated cirrhosis. To date, the correlated risk factors for severe liver events among inpatients are unknown. They may be much different from outpatients under long-term ART. Therefore, we carried out a retrospective study using multiple liver tests parameters to clarify the correlated risk factors of severe liver events in order to make rapid diagnoses and initiate prompt treatment.

## Method

### Study Design and Patients Enrollment

We conducted a retrospective study of inpatients who had already been diagnosed with HIV infection and were first admitted to the Department of Infectious Disease at the First Affiliated Hospital of China Medical University (Shenyang, China) from January 1, 2010, to December 31, 2019. Both cases with liver injury on admission and individuals without liver injury on admission but developing new liver injury during hospitalization were recruited. Exclusion criteria included: (1) patients with sustained normal liver function tests at admission and during the hospitalization; (2) patients whose ALT measurements at baseline were not available; (3) patients whose elevated ALP was due to skeletal injury; (4) patients whose elevated TBIL was due to hemolysis or extrahepatic bile duct obstructive diseases; and (5) patients with myositis. The Clinical Research Ethics Committee approved this study of the First Affiliated Hospital of China Medical University.

### Definitions of Outcomes and Covariates

Clinical data, including demographic data, underlying medical conditions, liver tests and clinical course, were obtained from patients' medical records, which were accomplished by three authors simultaneously. We separately analyzed the factors that might influence severe liver damage.

Liver injuries were defined as participants who had at least one incident elevation of ALT, aspartate aminotransferase (AST), ALP, or TBIL above the upper limit of normality (ULN). Severe liver injury in our study was defined as individuals who had plasma ALT, AST, or ALP values > 5 times above the ULN or TBIL values > 3 times above the ULN, which was according to at least grade 3 in commendations on management of immune-mediated liver injury induced by immune checkpoint inhibitors (14); otherwise, they were defined as mild or moderate liver injury. Cholestasis was defined as individuals with ALP values > 1.25 times the ULN and GGT > 3 times the ULN. Alcohol consumption was defined as patients who drank more than 40 grams (20 grams for female patients) of alcohol per day for more than 5 years (15). Cases of DILI were identified, assessed for a causality, categorized based on the updated Roussel Uclaf Causality Assessment Method (RUCAM) and DILI guidelines (14, 16, 17) and the exclusion of other liver diseases. HCV infection was defined as individuals who were both HCV- antibody positive and HCV-RNA positive. The resolved liver injury was defined as partial or complete restoration of the profile of liver tests when discharged compared to the worst profile during hospitalization. Hyponatremia was described as a serum sodium concentration <135 mmol/L. Hyperlipidemia was defined as serum triglycerides, total cholesterol, or low-density lipoprotein above the ULN (18). Thrombocytopenia was described as a platelet count <100,000/μL. The World Health Organization (WHO) clinical stage of all patients was based on the most severe clinical stage in their medical history records. Ultrasound was the diagnostic procedure for fatty liver.

### Statistical Analysis

The results were expressed as numbers, medians (interquartile ranges), and percentages. We compared conditions between patients with mild or moderate liver damage and patients with severe liver damage. The means for continuous variables were compared using the Student's *t*-test for normally distributed data. Otherwise, the Mann-Whitney U test was used. Proportions for categorical variables were compared using the chi-squared test. Fisher's exact test was used when the data were sparse. Univariate and multivariable logistic regression models were employed to assess factors associated with severe liver damage, with odds ratios (OR), adjusted odds ratios (aOR), and 95% confidence intervals (CI). After assessing the *P*-value from the univariate model, variables with *P* < 0.1 were introduced into multivariable logistic regression models. *P*-values < 0.05 were considered to be statistically significant for all cases. All analyses were performed by using SPSS software for Windows version 22.0 (Chicago, IL).

## Result

### Clinical Characteristics at Baseline

Among 708 patients who had baseline liver tests, three patients were first excluded, including one patient with elevated ALP due to skeletal injury and two patients with myositis. Among the remaining 705 patients, 212 had sustained normal liver function tests at admission and during the hospitalization, while 493 had abnormal liver tests levels. The percentage of severe liver events among all inpatients was 8.9% (63/705). The rate of severe liver events among inpatients with abnormal liver tests was 12.8% (63/493).

**Figure 1:**
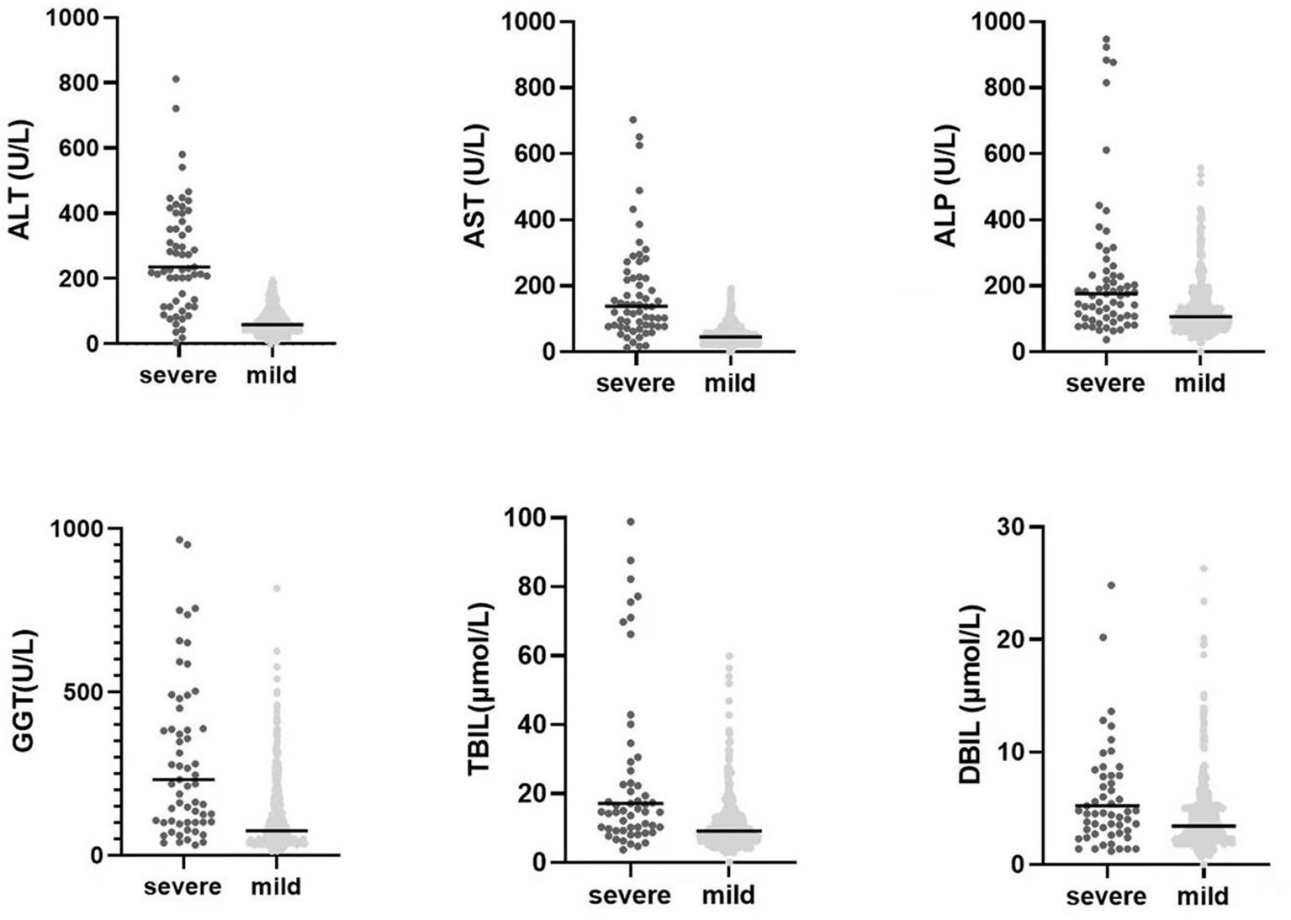
The comparision of liver function tests levels between mild or moderate liver injury HIV-infected inpatients and severe liver injury HIV-infected inpatients. The *P*-values of all of six parameters were <0.05 with statistically significant by using the Mann Whitney U test.

A total of 493 patients with liver damage were enrolled in this study. A total of 441 cases (89.5%) in our study were sexually transmitted, and 267 patients (54.2%) were men who had sex with men (MSM). The median age was 36 years (range: 20–79 years). There were three cases with compensated cirrhosis and six patients with decompensated cirrhosis. AIDS-related illnesses were still prominent problems for inpatients, including pneumocystis jirovecii pneumonia (PJP), TB, invasive fungal disease, malignancies, bacterial bloodstream infections, central nervous system (CNS) disease and disseminated mycobacterium avium complex (MAC) disease (Table 1). Among 493 patients with liver damage, a total of 49 patients died in the hospital. The in-hospital mortality rate was 9.9%. Non-liver-related death accounted for 98.0% of deaths. The direct causes of death included PJP (20), TB (16), sepsis syndrome (3), malignant tumors (2), cryptococcal meningitis (3), invasive pulmonary aspergillosis infection (3), HIV encephalopathy (1) and hepatic failure (1). The liver function tests levels of patients with severe liver injury compared with mild or moderate-liver injury patients were presented in Figure 1. Other common laboratory test abnormalities were summarized in Table 1.

**Table 1:** Baseline clinical and laboratory characteristics of 493 hospitalized HIV patients.

**Data**	**No. (%)**
Age (years) ≤ 40	291 (59.0%)
> 40	202 (41.0%)
Male	465 (94.3%)
BMI < 18.5 kg/m^2^	383 (77.7%)
Alcohol consumption history	68 (13.8%)
Hyperlipemia	110 (22.3%)
Diabetes	26 (5.3%)
Hypertension	17 (3.4%)
ART prior to admission	236 (47.9%)
NSAID exposure > 7 days before admission	309 (62.7%)
Cotrimoxazole exposure	241 (48.9%)
ATDs exposure	213 (43.2%)
Anti- fungal drugs exposure	31 (6.3%)
HBV coinfection	53 (10.8%)
HCV coinfection	15 (3.0%)
NAFLD	21 (4.3%)
ALD	17 (3.4%)
WHO clinical stage III-IV	434 (88.0%)
PJP	241 (48.9%)
TB	172 (34.9%)
CNS infection	66 (13.4%)
Malignant tumor	33 (6.6%)
Invasive fungal infection	31 (6.3%)
Bacterial bloodstream infection	29 (5.9%)
Cytomegalovirus retinitis	38 (5.3%)
Disseminated Mycobacterium avium complex disease	9 (1.9%)
CD4 T counts < 200 (/μL)	403 (81.7%)
CRP (>10 mg/L)	356 (72.2%)
Albumin (<30 g/L)	218 (44.2%)
PT > 13.7 s	205 (41.6%)
Hyponatremia	197 (40.0%)
HB (<9 g/L)	58 (11.8%)
Thrombocytopenia	48 (9.7%)
Scr (>104 μmol/L)	5 (1.0%)

*ART, antiretroviral therapy; BMI, body mass index; ATDs, anti-tuberculosis drugs; HBV, hepatitis B virus; HCV, hepatitis C virus; ALD, alcoholic liver disease; NAFLD, non-alcoholic fatty liver disease; PJP, pneumocystis jiroveci pneumonia; TB, tuberculosis; CNS, central nervous system; HIV, human immunodeficiency virus; CMV, cytomegalovirus; CRP, C-reactive protein; ALT, glutamic alanine transaminase; HB, hemoglobin; Scr, serum creatinine*.

Compared with the patients with mild or moderate liver damage, the patients with severe liver damage were more likely to have anti-tuberculosis drug (ATD) application (49.2 vs. 32.8%, *p* =0.037), cotrimoxazole exposure (65.1 vs. 46.5%, *p* = 0.010), viral hepatitis (26.9 vs. 11.8%, *p* < 0.001), a history of alcohol consumption (25.4 vs. 12.1%, *p* = 0.004) and thrombocytopenia (20.6 vs. 8.1%, *p* = 0.002) (Table 2).

**Table 2:** Baseline characteristics of patients with severe liver injury compared with mild or moderate-liver injury patients.

**Characteristics**	**Severe liver injury [*n* (%)]**	**Mild or moderate liver injury [*n* (%)]**	***P***
Total	63 (100)	430 (100)	
Age, years [median (IQR)]	37 (32, 50)	36 (29, 48)	0.267
Age > 40 years	29 (46.0)	173 (40.2)	0.382
Male	61 (96.8)	404 (93.9)	0.358
BMI (kg/m^2^)			0.615
<18.5	50 (79.4)	333 (77.4)	
18.5–23.9	2 (3.2)	27 (3.2)	
≥24	11 (17.5)	70 (16.3)	
Underlying medical condition	14 (22.2)	58 (13.5)	0.067
WHO clinical stage III-IV	53 (84.1)	381 (88.6)	0.307
Alcohol consumption history	16 (25.4)	52 (12.1)	0.004^#^
ART prior to admission	34 (53.9)	202 (46.9)	0.300
ART regimen			0.752
NVP-containing ART regimen	22 (34.9)	137 (31.9)	
Non-NVP-containing ART regimen	23 (36.5)	150 (34.9)	
Without ART	18 (28.6)	143 (33.3)	
Hospital stay days [median (IQR)]	14 (10, 20)	13 (8, 18)	0.227
Hospital mortality	8 (12.7)	41 (9.5)	0.433
CD4 < 200 (/μL)	55 (87.3)	348 (80.9)	0.222
Hyperlipidemia	16 (25.4)	94 (21.8)	0.529
Viral hepatitis	17 (26.9)	51 (11.8)	<0.001^#^
ALD or NAFLD	5 (7.9)	33 (7.6)	0.942
Thrombocytopenia	13 (20.6)	35 (8.1)	0.002^#^
Hyponatremia	20 (31.7)	177 (41.2)	0.154
CRP > 10 mg/L	40 (63.4)	316 (73.5)	0.098
Cotrimoxazole exposure	41 (65.1)	200 (46.5)	0.010^#^
Cotrimoxazole before admission	10 (15.8)	34 (7.9)	
Cotrimoxazole after admission	31 (49.2)	166 (38.6)	
ATDs exposure	35 (49.2)	178 (32.8)	0.037^#^
ATDs before admission	13 (20.6)	57 (13.3)	
ATDs after admission	22 (34.9)	121 (28.1)	
Anti- fungal drugs exposure	6 (9.5)	25 (5.8)	0.258
NSAID exposure >7 days before admission	38 (60.3)	271 (63.0)	0.679
HB < 9 g/dL	5 (8.0)	53 (12.3)	0.783
ALB < 30 g/L	28 (44.4)	190 (44.2)	0.969
PT > 13.7 s	27 (42.9)	178 (41.4)	0.826
Scr > 104 mmol/L	1 (1.6)	4 (0.9)	0.627
CHE < 4,000 (U/L)	13 (20.6)	99 (23.0)	0.673

# *P-value < 0.05, statistically significant with the use of chi-square test or Fisher's exact test*.

*IQR, interquartile range; BMI, body mass index; WHO, World Health Organization; ART, anti-retroviral therapy; NVP, nevirapine; ALD, alcohol-related liver disease; NAFLD, non-alcoholic fatty liver disease; CRP, C-reactive protein; ATDs, anti-tuberculosis drugs; NSAIDs, non-steroidal anti-inflammatory drugs; HB, hemoglobin; ALB, albumin; PT, prothrombin time; Scr, serum creatinine; CHE, cholinesterase*.

### Treatment Adjustment After Severe Liver Damage

Previous drugs should be modified when severe liver damage occurs. Drugs with hepatotoxicity should be avoided. Liver protective drugs, such as silymarin capsules, ursodeoxycholic acid capsules, bicyclol tablets, isoglycyrrhizinate magnesium injections, S-adenosylmethionine injections and L-glutathione injections are usually selectively used.

### Factors Associated With Severe Liver Injury

Among 493 patients with abnormal liver parameters, 213 cases presented with liver injury at the baseline visit. At the same time, 280 cases developed new liver damage during hospitalization. Resolved liver injury was observed in 100% of 430 cases with mild or moderate grades of liver injury. Among 63 patients with severe liver injury, 32 cases met the definition of severe liver injury on admission, and 31 cases developed severe liver injury during hospitalization. Twenty-six patients with severe liver injury met the criteria of cholestasis. Among patients with severe liver injury, 51 cases (81.0%) resolved when discharged. Among 12 cases with unresolved or worsened liver injury, one patient died in the hospital due to hepatic failure.

The direct causes of severe liver injury in 63 cases included DILI (43), viral hepatitis (12), sepsis syndrome (4), CMV (2), EBV (1), and malignant tumor (1). Six patients met the criteria of liver failure, including subacute liver failure (5) and chronic liver failure (1). Among cases with severe liver injuries, DILI was identified and scored in 68.3% (43/63) patients including 3 cases with high probable DILI, 36 cases with probable DILI, 4 cases with possible DILI. The pattern of DILI was hepatocellular in 36.5% (23/43) of patients, cholestatic in 25.6% (11/43) of patients, and mixed in 20.9% (9/43) of patients. Nine patients with DILI had fever and eruptions. One patient with nevirapine (NVP)-DILI presented with rash with eosinophilia and systemic symptoms (DRESS). Cases with DILI were caused by ATDs (11), cotrimoxazole (10), NVP (10), other antibiotics (5), efavirenz (4), and non-steroidal anti-inflammatory drugs (NSAIDs) (3). Hepatitis virus co-infection accounted for 19.0% (12/63) of cases with severe liver injury, while five cases met the criteria for IRIS. Among six patients with cirrhosis in severe liver damage group, there were 5 cases at Child-Pugh class B and one case at Child-Pugh class C. Only one patient was aware of his HBV-infected status prior to HIV diagnosis. Among 63 patients with severe liver injury, seven patients died in the hospital, including six patients with non-hepatic-associated death and one patient with hepatic-associated death.

Data with a *P*-value < 0.1 in the univariate analysis were entered into the multivariate logistic regression model, including exposure to ATD (*P* = 0.012), cotrimoxazole exposure (*P* = 0.007), viral hepatitis (*P* = 0.004), alcohol consumption history (*P* = 0.005), and thrombocytopenia (*P* = 0.007). Finally, analyses using the multivariate logistic regression model showed that exposure to ATD (aOR = 1.835, 95% CI: 1.031–3.268; *P* = 0.039), cotrimoxazole exposure (aOR = 2.775, 95% CI: 1.511–5.096; *P* = 0.001), comorbidity of viral hepatitis (aOR = 2.340, 95% CI: 1.161–4.716; *P* = 0.017), alcohol consumption history (aOR = 2.392, 95% CI: 1.199–4.769; *P* = 0.013) and thrombocytopenia (aOR = 2.583, 95% CI: 1.127–5.917; *P* = 0.025) were risk factors for severe liver injury (Table 3).

**Table 3:** Factors associated with severe liver injury among HIV-infected inpatients in Shenyang.

**Factors**	**OR (95%CI)**	**P1**	**Adjust OR (95%CI)**	**P2**
**Viral hepatitis**				
No	1.000			
Yes	2.530 (1.336–4.789)	0.004	2.340 (1.161–4.716)	0.017
**Cotrimoxazole exposure**				
No	1.000			
Yes	2.143 (1.235–3.720)	0.007	2.775 (1.511–5.096)	0.001
**Anti-TB drugs exposure**				
No	1.000			
Yes	1.986 (1.165–3.385)	0.012	1.835 (1.031–3.268)	0.039
**Thrombocytopenia**				
No	1.000			
Yes	2.709 (1.320–5.557)	0.007	2.583 (1.127–5.917)	0.025
**Alcohol consumption history**				
No	1.000			
Yes	2.475 (1.309–4.679)	0.005	2.392 (1.199–4.769)	0.013

## Discussion

In this study, the percentage of severe liver events among all inpatients was 8.9%, much higher than the 0.3% of ART-naïve studies (12). Among cases with severe liver damage, DILI was the predominant finding (68.3%), followed by hepatitis virus co-infection (19.0%). PJP and TB were two main coexistent OIs among inpatients. The post-exposure monitoring of cotrimoxazole and ATD should be recommended. The presence of thrombocytopenia was a high-risk factor for the occurrence of severe liver injury. Although most liver tests abnormalities resolved over time, they were not completely reversible in a small number of cases.

The acute DILI in our study occurred mainly within the first 3 months after taking drugs. The early-onset (within 12 weeks) DILI with a rash or fever were associated with hypersensitivity reactions, which showed a higher prevalence in HIV-infected individuals with advanced immunodeficiency than in the general population (19, 20). Non-nucleoside reverse transcriptase inhibitors, such as NVP and efavirenz (EFV), showed a high possibility of liver injury (2, 21). NVP was associated with non-specific hepatitis, while EFV was associated with submassive necrosis (7). Compared to EFV, NVP showed more frequent severe hepatic injury in our study. However, NVP-containing ART regimens were not a significant risk factor for severe liver damage due to its usual presentation of mild to moderate liver injury (22) and genetic polymorphisms (23). Luckily, new medications without apparent hepatotoxicity have become widely available. ATD were an important cause of DILI because one-third of patients had TB co-infection in our study. There were high incidence rates of DILI among cotreated HIV/TB co-infected patients (24). Although liver tests abnormalities were generally reversible within 4–6 weeks of discontinuation of the offending drugs, DILI caused by ATD was associated with high mortality (25). Hepatotoxicity was also associated with isoniazid preventive therapy (26). Among antibiotic-induced liver injuries, cotrimoxazole has the highest risk of cholestatic or ductopenic injury (7, 27). Approximately half of the inpatients were diagnosed with PJP in this study. Although NSAIDs are available and widely used for antipyretics in China, they are not associated with increased liver damage risk due to the lack of overdoses. The application of traditional Chinese medicine or herbal and dietary supplements is uncommon in the HIV-positive population. Apart from DILI, the updated RUCAM was also recommended for assessing herb-induced liver injury (28, 29).

Patients co-infected with HBV or HCV were prone to liver injury, identical to a previous study (30). HCV co-infection presented a high risk of developing severe liver toxicity related to NVP (31). An approximately 10.8% prevalence of HBV co-infection was found in our study. In comparison, the prevalence of HCV co-infection was only 3.0%. HBV co-infection was a more common factor associated with severe liver injury than HCV co-infection in our study. Most patients were unaware of their HBV-infected status at the time of HIV diagnosis. HBeAg-negative chronic HBV infection with a high HBV DNA load is an apparent characteristic (32). Hepatic flares were occasionally considered as hepatitis virus-associated IRIS (33). Compared to intravenous drug users and former plasma donors, most cases were sexually transmitted in our study, who might be less associated with HCV infection.

Our study indicated the importance of addressing previous alcohol use among inpatients, a high-risk population for severe liver damage. Patients with binge drinking were more often to experience a liver-related event, even liver-related death (34). Hazardous drinking is a significant risk factor for liver fibrosis, particularly among HIV-positive patients without HCV coinfection (35). The underlying mechanism is that alcohol metabolism potentiates HIV-induced hepatotoxicity (36). Notably, alcohol intake > 40 g per day was associated with severe liver toxicity in those patients receiving NVP-or EFV-containing regimens (37).

Thrombocytopenia was also a contributing factor for the occurrence of severe liver damage in this study. Thrombocytopenia is common in HIV-infected patients. Its seriousness and incidence are related to the stage of HIV infection, hypersplenism, portal hypertension and other mechanisms (38). As the common tools for assessing chronic liver disease (CLD), both aspartate aminotransferase-to-platelet ratio index (APRI) and fibrosis index based on four factors (FIB-4) could be affected by platelet counts. Therefore, evaluation for liver fibrosis should be performed among cases with thrombocytopenia, which would be a better combination with transient elastography.

Elevated ALP, GGT, and TBIL were common in cases with IRIS-associated liver injury (39). Liver injury associated with TB-IRIS is characterized by the infiltration of neutrophils, plasma cells, and abundant lymphocytes within granulomas (40). Liver biopsy of cases with HBV-IRIS showed lymphocytic infiltration, predominantly diffuse CD8+ T cell infiltration in the portal areas and lobules (41). Elevated TBIL was more common than elevated ALT in patients with decompensated liver cirrhosis or liver abscesses. We should note that most severe liver events in this study were associated with acute reversible liver damage in the absence of pre-existing CLD. Low serum ALB levels are prevalent laboratory test abnormalities in HIV-infected patients, irrespective of liver injury. Prolonged PT was sensitive but non-specific to acute or chronic liver failure. Abdominal ultrasound examination or computed tomography scans were recommended for routine screening among inpatients in our department. Although clinicopathological diagnosis is essential, liver biopsy is not always necessary in patients with acute reversible liver injury.

Our study is associated with some limitations. Only hospitalized patients were included in this analysis, and they lacked liver biopsies and follow-up studies after discharge. Inpatients outside the Department of Infectious Disease in our hospital were excluded in this retrospective study because there was a lack of intensified medical care strategy and professional assessment of HIV inpatients in other departments. Importantly, we could collect detailed information on potentially hepatotoxic medications and frequently monitor liver conditions using multiple variables among inpatients. Our data are only from our hospital, and further studies from multiple centers should be conducted and verified.

In conclusion, the severe liver injury event is not uncommon among HIV-infected inpatients. Patients with HBV co-infection and cotrimoxazole or ATD-induced DILI deserve special medical care and frequent monitoring of liver tests. Among patients with a history of alcohol consumption and thrombocytopenia, we should further assess liver fibrosis. These findings have important implications for identifying risky individuals who are more likely to develop severe liver injury and improve therapeutic regimens.

## Data Availability Statement

The raw data supporting the conclusions of this article will be made available by the authors, without undue reservation.

## Ethics Statement

The studies involving human participants were reviewed and approved by the Clinical Research Ethics Committee of the First Affiliated Hospital of China Medical University. The patients/participants provided their written informed consent to participate in this study.

## Author Contributions

SL was responsible for data collection, statistic analysis, and article writing. YZ, YWa, CL, WW, XL, and PL participated in the clinical diagnosis and treatment. YWe designed the article and took part in writing and revising. QH designed the study and was responsible for the statistical analysis. All authors contributed to the article and approved the submitted version.

## Funding

This work was supported by the Double First-Class University and Discipline Construction funds of China Medical University (3110119068 to YWe) and the Fund of National Natural Science (82073620 to QH). The funders had no role in the study design, data collection, and analysis.

## Conflict of Interest

The authors declare that the research was conducted in the absence of any commercial or financial relationships that could be construed as a potential conflict of interest.

## Publisher's Note

All claims expressed in this article are solely those of the authors and do not necessarily represent those of their affiliated organizations, or those of the publisher, the editors and the reviewers. Any product that may be evaluated in this article, or claim that may be made by its manufacturer, is not guaranteed or endorsed by the publisher.

## Abbreviations

HBV: hepatitis B virus
HIV: human immunodeficiency virus
HCV: hepatitis C virus
AIDS: acquired immune deficiency syndrome
FHF: fulminant hepatic failure
DILI: drug-induced liver injury
ART: antiretroviral therapy
ALT: alanine transaminase
AST: aspartate aminotransferase
RUCAM: Roussel Uclaf Causality Assessment Method score
OIs: opportunistic infections
MTB: Mycobacterium tuberculosis
ATDs: anti-tuberculosis drugs
NTM: non-tuberculosis mycobacteria
IRIS: immune reconstitution inflammatory syndrome
MSM: men who have sex with men
BMI: body mass index
CHE: cholinesterase
ALP: alkaline phosphatase
GGT: gamma-glutamyltransferase
TBIL: total bilirubin
DBIL: direct bilirubin
PT: prothrombin time
INR: international normalized ratio
ULN: upper limit of normality
WHO: World Health Organization
IQR: interquartile range
OR: odds ratio
aOR: adjusted odds ratio
CI: confidence interval
HB: hemoglobin
ALB: albumin
Scr: serum creatinine
NSAIDs: non-steroidal anti-inflammatory drugs
TB: tuberculosis
TMP-SMZ: trimethoprim-sulfamethoxazole
NVP: nevirapine
EFV: efavirenz
CRP: C-reactive protein
PJP: pneumocystis jiroveci pneumonia
CNS: central nervous system
MAC: mycobacterium avium complex disease
ALD: alcohol-related liver disease
NAFLD: non-alcoholic fatty liver disease
CMV: cytomegalovirus
HR: hazard ratio
APRI: the aspartate aminotransferase-to-platelet ratio index
FIB-4: the fibrosis index based on four factors
DRESS: eosinophilia and systemic symptoms
CLD: chronic liver disease.
